# Repeated superovulation increases the risk of osteoporosis and cardiovascular diseases by accelerating ovarian aging in mice

**DOI:** 10.18632/aging.101449

**Published:** 2018-05-22

**Authors:** Jinjin Zhang, Zhiwen Lai, Liangyan Shi, Yong Tian, Aiyue Luo, Zheyuan Xu, Xiangyi Ma, Shixuan Wang

**Affiliations:** 1 Department of Obstetrics and Gynecology, Tongji Hospital, Tongji Medical College, Huazhong University of Science and Technology, Wuhan, Hubei, People’s Republic of China; 2 Maternal and Child Health Hospital of Zigong, Sichuan, People’s Republic of China; 3 Department of Obstetrics and Gynecology, Hubei Maternity and Child Health Care Hospital, Wuhan, Hubei, People’s Republic of China; 4 The Central Hospital of Enshi Autonomous Prefecture, Enshi Autonomous Prefecture, Hubei, People’s Republic of China

**Keywords:** repeated superovulation, osteoporosis, cardiovascular disease, accelerated ovarian aging

## Abstract

Superovulation procedures and assisted reproductive technologies have been widely used to treat couples who have infertility problems. Although generally safe, the superovulation procedures are associated with a series of complications, such as ovarian hyper-stimulation syndrome, thromboembolism, and adnexal torsion. The role of long-term repeated superovulation in ovarian aging and especially in associated disorders such as osteoporosis and cardiovascular diseases is still unclear. In this study, we sought to determine if repeated superovulation by ten cycles of treatment with pregnant mare serum gonadotropin/human chorionic gonadotropin could affect ovarian reserve, ovarian function, bone density and heart function. Ovarian reserve and function were reflected by the size of the primordial follicle pool, anti-Mullerian hormone expressions, hormone levels and fertility status. Furthermore, we examined bone density and heart function by microCT and cardiovascular ultrasonography, respectively. After repeated superovulation, the size of the primordial follicle pool and the expression of anti-mullerian hormone decreased, along with the concentrations of estrogen and progesterone. Mice exposed to repeated superovulation showed an obvious decrease in fertility and fecundity. Furthermore, both bone density and heart ejection fraction significantly decreased. These results suggest that repeated superovulation may increase the risk of osteoporosis and cardiovascular diseases by accelerating ovarian aging.

## Introduction

More than 15% of couples will likely face infertility problems during their reproductive life, and by 2025, the number of women with this problem will be approximately seven million worldwide [1]. Superovulation procedures and assisted reproductive technologies (ART) have been widely used to treat couples who have infertility problems. Although generally safe, superovulation procedures can lead to serious complications, which include ovarian hyper-stimulation syndrome (OHSS), thromboembolism, and adnexal torsion [2]. In addition, a series of studies have suggested that repeated superovulation (RS) may influence the structure and function of the ovary. In mice, repeated ovarian stimulation can induce oxidative damage and mitochondrial DNA mutations in ovaries, increase the incidence of oocyte spindle defects, and decrease the quality of oocytes [3–5]. Clinical studies have found that ovulation induction cycles can impair the ovarian response and/or alter the quality of oocytes [6]. However, there is no consensus regarding the adverse effects of repeated ovulation stimulation. Some large-scale retrospective studies have shown that the number of oocytes retrieved over five repeated in vitro fertilization (IVF) cycles remained the same and that there was no difference in oocyte quality [7]. Therefore, although an increasing number of studies have focused on the effects of repeated ovulation on ovaries or oocytes, studies conducted to date have failed to reach a definitive conclusion as a result of numerous shortcomings, such as inconsistent drug exposure and use and relatively short follow-up periods [8]. Furthermore, ovarian damage often causes a decline in follicular quantity and quality, which is a complex process associated with ovarian aging. Changes in hormone production caused by ovarian aging may affect various health consequences including vasomotor symptoms, cardiovascular diseases (CVDs), osteoporosis, cognition, depression, mood disorders, sexual function, and vaginal atrophy [9]. Until now, few studies have focused on long-term effects of RS on health outcomes, specifically ovarian aging and aging-associated disorders such as osteoporosis and CVD in mouse models, which is of vital importance.

In the normal ovulation cycle, FSH and LH collaboratively regulate follicular growth, oocyte maturation, ovulation, corpus luteum formation and steroid hormone secretion. Pregnant mare serum gonadotropin (PMSG) has the activity of both FSH and LH, but priority is given to the role of FSH. Therefore, PMSG has obvious effects of stimulating follicle development and can promote ovulation and luteum formation [10]. Human chorionic gonadotropin (hCG) has the effect of luteinizing hormone, which can stimulate ovulation to near follicle maturation [11]. Exogenous gonadotropins, such as PMSG and hCG, are often used to induce superovulation in humans and rodents to induce estrus, ovulation, and coitus and an increase in follicle numbers, thus improving pregnancy success. Follicular stimulation in women was attempted as early as 1931 using PMSG [12]. The repeated PMSG/hCG administration protocol is well-established to induce superovulation in mice for almost 30 years in many laboratories [13,14], and at least 5 cycles of ovulation are used. It is worth noting that many women have to receive several rounds of superovulation to become pregnant throughout the treatment process. Smith [15] reported that the cumulative prognosis-adjusted live-birth rate across all repeated ovulation cycles continued to increase up to the ninth cycle, with 65.3% of women who are undergoing IVF achieving a live birth by the sixth cycle in the United Kingdom. These findings support the efficacy of extending the number of IVF cycles beyond 3 or 4. In some countries or regions, such as Israel, many women may even have 12-20 repeated ovulations due to insurance policies [16]. Is there a limit to the number of repeated ovulation cycles for an individual patient?

Thus, in this article, we discuss long-term RS with a focus on changes in ovary and heart function and bone density. Our study used mice as the study model to evaluate the structure and function of ovaries that have previously received 10 cycles of RS treatments. We believe that this model can remove the influence of other factors, determine whether repeated ovulation stimulations have negative effects on the ovaries, and investigate the potential associated underlying mechanisms.

## RESULTS

### Repeated superovulation decreased the physical strength of mice

**Figure 1:**
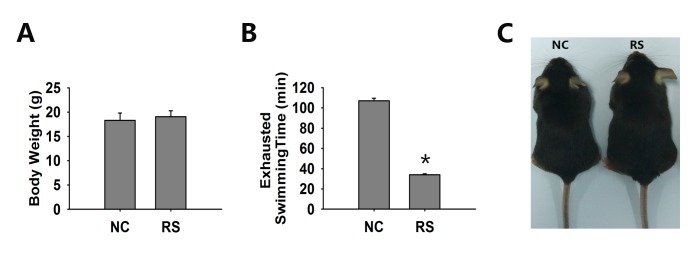
**The general condition of both groups of mice.** (**A**) Body weight was not significantly different in both groups. (**B**) The exhausted swimming time of the RS mice was markedly decreased compared to that of the NC group. (**C**) Representative photos of NC and RS group mice. *significantly greater at *P*<0.05 compared to the NC group.

We evaluated the general conditions and physiological function of the two groups of mice. The outcomes of the physiological parameters were analyzed and are summarized in Fig. 1. The body weight of the RS group did not increase significantly after ten cycles of RS (Fig. 1A). We used the exhausted swimming time to represent physical strength and found that the strength of the RS group mice was markedly decreased (Fig. 1B). A photo of the two groups of mice is shown in Fig. 1C.

### Repeated superovulation reduced the bone density of mice

**Figure 2:**
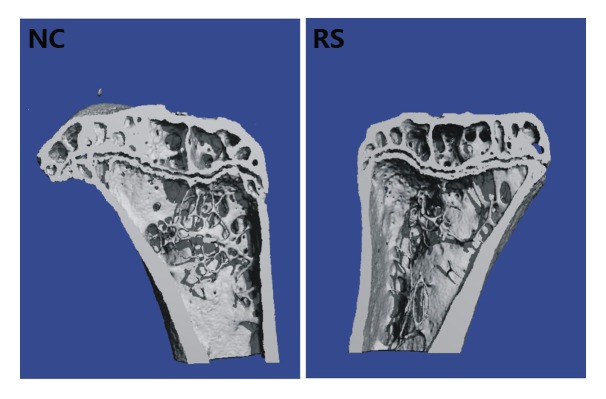
**The architecture of trabecular bone of the left tibia in the NC group and RS group.** The trabeculae separation was outstandingly increased in the tibia of the RS group mice compared with that of the NC group.

Osteoporosis, which is characterized by changes in the architecture of bone, is the most common consequence of menopause. We examined the bone mass to evaluate the effect of the ovary in the repeated super-ovulated mice on the skeletal system. Six months after modeling, the architecture of bone in each group was investigated using the SCANCO Micro-CT50 (SCANCO Medical) equipped system through trabecular bone analysis (Fig. 2, Table 1). The trabecular bone volume (BV), number of trabeculae (TbN), connectivity density (ConnD), trabeculae thickness (TbTh) and structure model index (SMI) in the tibia showed no significant differences between the two groups. However, the trabeculae separation (TbSp) was outstandingly increased in the tibia of the RS group mice compared with that of the NC group. These changes agreed well with the observed reduced bone mineral density (BMD), which is directly related to the material stiffness of the bone.

**Table 1:** Trabecular parameters of proximal tibia.

Variable	NC	RS
BV(mm^3^)	1.40±0.06	1.42±0.14
Tb.N.(1/mm)	4.98±0.21	4.30±0.11
Tb.Th.(mm)	0.08±0.00	0.08±0.00
Tb.Sp(mm)	0.25±0.01	0.30±0.01*
ConnD(1/mm^3^)	312.62±55.45	245.63±59.45
SMI	-2.62±0.25	-2.55±0.33

N=5 per group. Values are the means (±SD). RS: repeated superovulation group; BV, trabecular bone volume; TbN, number of trabeculae; TbTh, trabeculae thickness; TbSp, trabeculae separation; ConnD, connectivity density; SMI, structure modeli index.

### Repeated superovulation decreased the heart function of mice

**Figure 3:**
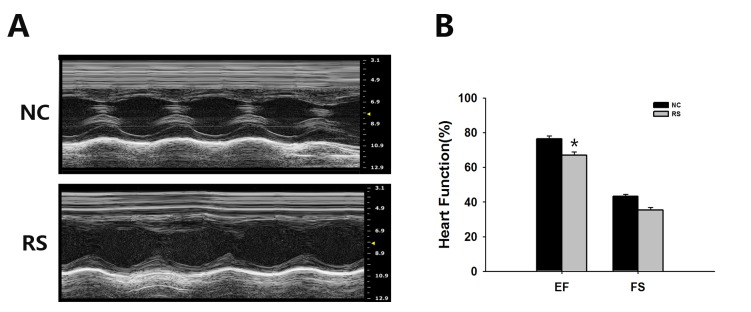
**Ultrasound imaging planes analysis used for cardiovascular assessments of mice of the NC and RS groups.** (**A**) The cardiovascular ultrasound imaging of the NC and RS groups. (**B**) Cardiac functions of the two groups were estimated by EF and FS. The EF in the RS group was decreased significantly compared with NC group.

Gender differences exist in cardiovascular disease risk, beyond the protective effect of estrogens, mostly burdening postmenopausal females [17]. Mortality rates of cardiac vascular disease remain higher in women than in men despite the increased awareness of cardiovascular risk factors over recent decades. Estrogens have a protective role in this regard in premenopausal women. However, after the decrease in estrogen levels, women are exposed to the same, or even higher, cardiovascular risks as males [18,19]. Because of this, we examined the echocardiography of the mice, especially the ejection fraction (EF) and fractional shortening (FS), which are the two most commonly used parameters for estimating cardiac function, in the two groups using a Vevo®1100 Imaging system. The representative ultrasonic cardiogram reflected left ventricular function of the heart in both groups of mice (Fig. 3A). Our results indicated that ventricular systolic function declined in the RS group mice. We also found that EF significantly decreased in the RS group compared with the NC group (Fig. 3B). No significant difference in FS was observed between the groups of mice.

### Repeated superovulation diminished the ovarian reserve

We further tested the effect of RS on the ovarian reserve and the size of primordial follicle pool. The expression level of AMH, which was only produced by the granulosa cells of primary, preantral and small antral follicles, could represent the number of growing follicles [20]. AMH has been demonstrated to be an ideal marker of ovarian reserves [21–25]. In addition to AMH, the ovarian primordial follicle pool, which serves as a source of developing follicles, is another ideal parameter to evaluate the ovarian reserve [26,27]. Therefore, in this study, we used both the AMH expression and numbers of primordial follicles to reflect the ovarian reserve, evaluating the effect of RS on the ovarian follicular quantity.

**Figure 4:**
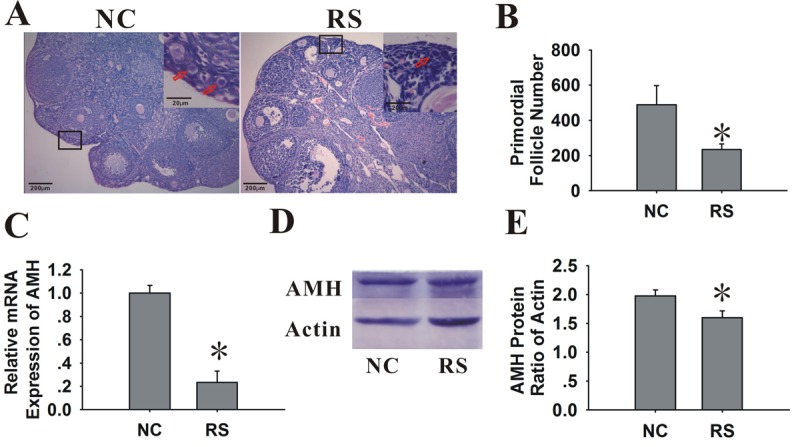
**Effects of RS on the ovarian reserve of mice.** (**A**) Photographs of the glass slides stained with HE in NC and RS mouse ovaries. Inset (upper right corner of each group) shows the number of primordial follicles (primordial follicles, red arrows). The images shown are at an original magnification of x200; insets are x400. (**B**) Bar graphs showed that the primordial follicle numbers of RS mice were significantly decreased compared to NC mice. (**C**) The mRNA expression of AMH decreased in RS mice. (**D-E**) The protein expression of AMH was determined using western blot analysis; bar graphs showed that the protein expression levels of AMH in RS mice were decreased compared to NC group.

When analyzing the glass slides that were stained with HE (Fig. 4A, B), we observed that the primordial follicle numbers in the mice treated with RS were lower than those in NC group. Compared with the NC group, the total AMH mRNA and protein expression of the ovaries in treated mice demonstrated a marked decline (Fig. 4C-E).

### Repeated superovulation weakened the ovarian endocrine and reproductive function of mice.

With the evidence that repeated ovarian stimulation reduced the ovarian reserve, we further evaluated ovarian function, including the estrous cycle length, serum estradiol and progesterone levels and reproductive capacity.

**Figure 5:**
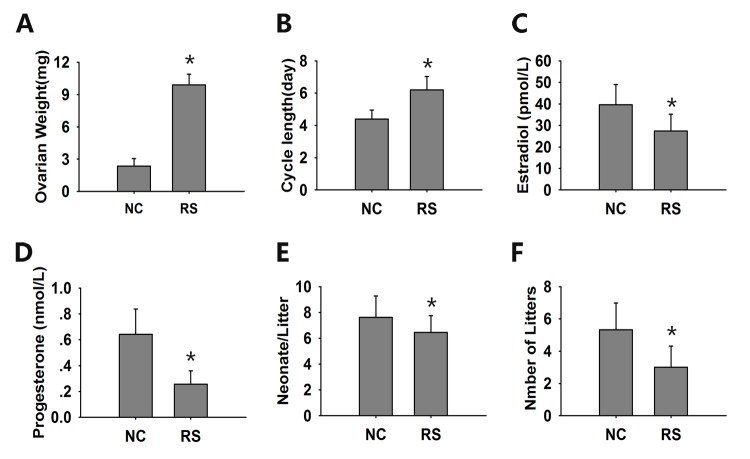
**The evaluation of ovarian endocrine and reproductive function.** (**A**) The ovarian weight of the RS group mice increased significantly compared with the NC group mice. (**B**) The estrous cycles of the RS mice were obviously prolonged. The concentrations of estradiol (**C**) and progesterone (**D**) were determined in duplicate using an EIA kit. The mean litter size (**E**) and the mean number of litters (**F**) were analyzed during a 6-month course of breeding. The RS mice displayed lower levels of hormones and reduced litter sizes and numbers compared to the NC group. Data presented are the mean ± SEM. * *P*<0.05 compared to the NC.

After repeated ovulation, the ovarian weight increased significantly (Fig. 5A). The estrous cycle is a direct manifestation of ovarian function. Fourteen days after the final hCG administration, all of the treated mice were restored to their independent estrous cycles. The estrous cycles of the RS mice were obviously prolonged when compared with those of the NC group (Fig. 5B).

The sex hormone levels of 17β-estradiol and proges-terone, which are secreted by granulosa and luteal cells, are usually used to evaluate ovarian endocrine function. We found that the serum levels of 17β-estradiol significantly decreased after RS with a concomitant decrease in progesterone levels (Fig. 5C, D).

As the decline in fertility and fecundity is the most obvious event of ovarian aging, we analyzed the mean litter size and mean number of litters of both groups after treatment during a 6-month course of breeding. Both the neonatal numbers per litter and the total litter numbers were remarkably decreased in the RS group compared with the NC group (Fig. 5E, F).

### Repeated superovulation affected the ultrastructure of follicles in the mice ovaries

**Figure 6:**
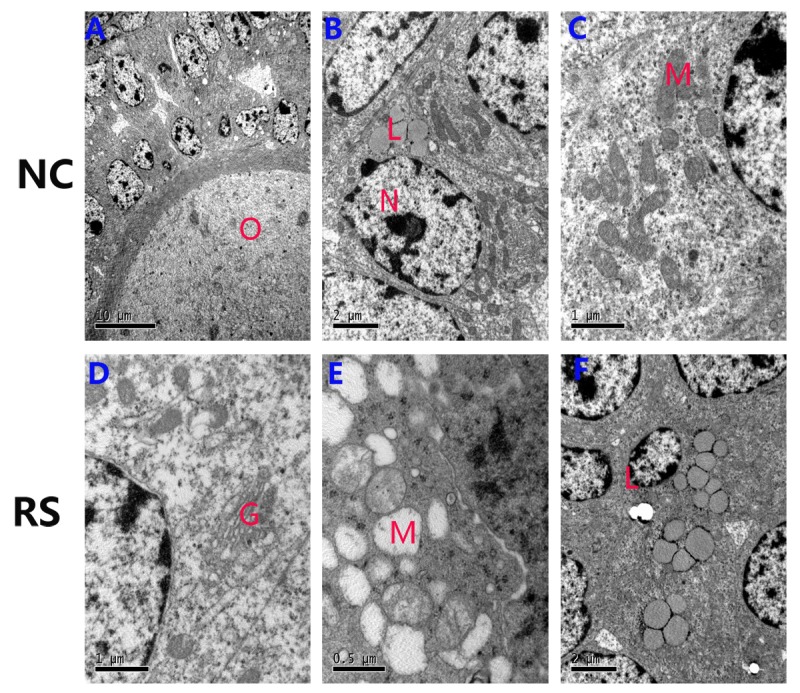
**Ultrastructural changes of the ovarian granulosa cells in both groups.** (**A-C**) The normal ultrastructure in the ovaries of NC mice. Few lipids were observed in the ovaries, and most mitochondria structures were normal. (**D-F**) The mitochondria were swollen, exhibited decreased matrix density, and developed flocculent dense bodies in the matrix space in the RS group mice. The number of lipid droplets and swollen Golgi complexes were increased in RS group mice. (O: oocyte; N: nucleus of granulosa cells; M: mitochondrial; L: lipid; G: Golgi complex).

To analyze the impact of RS on organizational structure, we viewed ultrastructural changes of the ovaries using electron microscopy (Fig. 6A-F). Analysis of the two groups indicated that the mean areas of the GC and GC nuclei were not significantly different (Table 2). However, a marked difference was observed between the NC and RS groups when mitochondrial morphology was compared. In the NC group, cristae were clearly visible and appeared tubular, whereas in most cells of the RS group, the cristae morphology could not be easily identified because highly electron- dense matrix and cristae were barely visible. Mitochondria in some cells became swollen and showed vacuolization and degeneration of the cristae and matrix in the RS group. Moreover, the amounts of lipid droplets and swollen Golgi apparatuses increased significantly in the RS group.

**Table2:** Ultrastructure morphology comparison of GCs.

Variable	NC	RS
Mean area GCs (μm2)	119.7±5.6	124.8±6.6
Mean area GC nuclei(μm2)	23.6±2.3	35.2±3.3
Mean nucleus/cell ratio	0.21	0.28
Cytoplasmic fraction of		
Mitochondria (%)	14.1±5.2	11.4±8.6
Lipid droplets (%)	26.0±4.5	11.7±4. 6*
Cells with defective mitochondria	4.7%	25.3%

### Repeated superovulation increased oxidative stress in the ovaries

**Figure 7:**
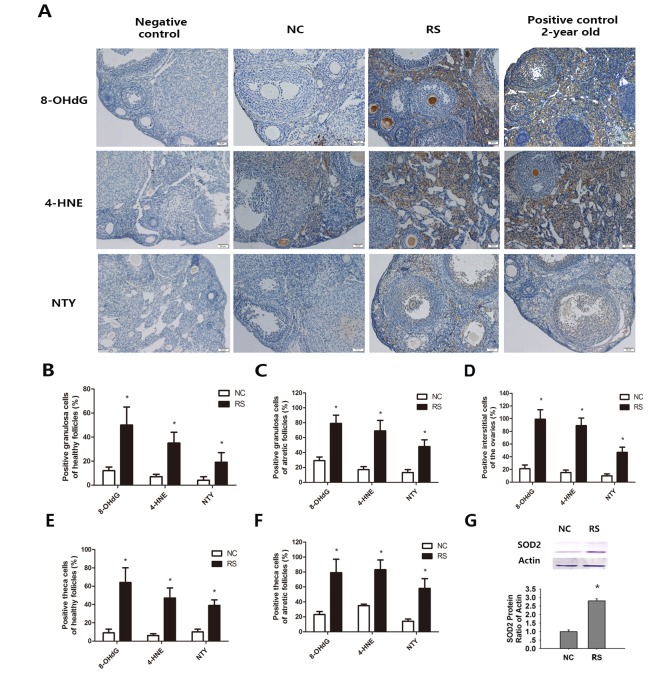
**Effects of repeated superovulation on oxidative damage and antioxidant enzymes in both groups.** Oxidative stress related oxidative damage in interstitial cells was scored using immunostaining for oxidative damage markers, as described in the Methods section. (**A**) Representative images of immunostaining (dark brown) for each oxidative damage marker in interstitial cells: Oxidative DNA damage (8-OHdG), lipid peroxidation (4-HNE) and protein oxidation (NTY) in the ovaries of the NC group, RS group and negative control and positive control groups. All original magnifications are x200. (**B-C**) The percentages of positive stained granulosa cells in healthy or atretic follicles. (**D-E**) The percentages of positive theca cells in the healthy or atretic follicles. (**F**) The percentages of positive interstitial cells in the ovaries. (**G**) The protein expression of the antioxidant gene SOD2 was significantly increased in the RS group. Values are expressed as the mean ± SEM. *P<0.05 compared to the NC group.

Oxidative lipid (4-HNE), protein (NTY), and DNA (8-OHdG) damage in interstitial cells and follicles of super-ovulated mice were measured. These molecules are widely accepted as biomarkers of oxidative DNA, protein, and lipid damage, respectively, in biological systems [28]. We observed that the expression levels of 8-OHdG, 4-HNE and NTY were all increased in the ovarian interstitial cells of RS mice (Fig. 7A). The percentage of follicles with positive staining for the indicated marker in the granulosa cells or theca cells of healthy and atretic follicles was significantly increased compared with the NC group (Fig. 7B-E). Oxidative damage markers in interstitial cells also increased when compared with the NC group mice (Fig. 7F). SOD2 is one of the most important antioxidant enzymes in all tissues due to its role in defense against oxidative stress, and its expression was upregulated when oxidative stress occurred. Analysis of the expression of this enzyme in both groups showed that repeated ovulation markedly increased the ovarian expression of this enzyme (Fig. 7G).

## DISCUSSION

Ovarian stimulation with exogenous gonadotropins and ovulation induction using hCG could cause OHSS, which is a potentially life-threatening iatrogenic complication. The clinical manifestations of OHSS are ascites and edema [29]. Our data showed that the body weights of the RS group did not significantly increase, and no evidence of ascites or edema was shown. The ovarian weight and size of the RS mice increased, which may be due to the increased number of recruited follicles. An exhaustive swimming exercise was used to test physical strength, and we found that physical strength was markedly decreased in the repeatedly super-ovulated mice.

Ovarian aging is characterized by a progressive decline in fertility attributed to the gradual depletion of the ovarian follicle pool and a gradual decrease in follicle quality because of accumulated damage in the ovary. The most notable characteristic of ovarian aging is the gradual loss of the primordial follicle pool [30]. We found that the number of primordial follicles in the RS group mice decreases after one month of superovulation. With the decrease in the number of antral follicles with age, the AMH serum levels declined and invariably became undetectable near menopause [31]. AMH was proven to be an excellent marker of ovarian reserve [21]. In our results, the AMH expression levels of the RS group decreased compared with the NC group. Our results indicated that repeated ovulation decreased the ovarian reserve.

Along with the decrease in follicle number, the decrease in follicle quality is the other remarkable characteristic of the ovarian aging process. Factually, our study showed that RS not only reduced the follicle number but also induced oxidative stress in the ovaries, which had also been reported previously [32]. Jinhwan Lim’s study revealed significant age-related increases in oxidative damaged DNA, protein and lipids in ovarian interstitial tissue, and these changes are markers of age-related changes due to oxidative damage in the ovary [28]. According to previous studies [3,33], we observed the amounts of increased products of oxidative damage, reflected by increased 8-OHdG, 4-HNE and NTY, in the ovaries. Increasing levels of endogenous ROS lead to a wide range of oxidative damage in cell structures, including ovarian quality. The alterations in the expression of biomarkers of oxidative damage suggested that mice induced by repeated ovulation could increase the oxidative damage in the ovaries, which is proposed to be an underlying factor of the aging process [34]. Moreover, the amounts of swollen mitochondria, Golgi's apparatuses and lipid droplets increased significantly in the RS group. These results indicated that follicle quality decreased in super-ovulated mice.

Menstrual changes are considered an early manifestation of ovarian dysfunction. During the menopausal transition, menstrual cycle regularity is progressively lost due to insufficient numbers of FSH-sensitive follicles that are present at any time in the ovaries [35,36]. The cycles of older mice became lengthened, and then they lose the capacity for cyclicity. Most of the vaginal lavages from acyclic mice were leukocytic (i.e., diestrus or metestrus-2) [37]. In our study, the length of the ovarian cyclicity of RS mice was obviously extended, which was paralleled with decreases in the follicle number. Current evidence suggests that the estradiol level decreases relatively late in the process of ovarian aging, and the plasma concentration of progesterone does not change significantly [38–40]. Our results showed that the plasma concentration of estradiol and progesterone decreased in the RS group mice. These results indicated that the ovarian endocrine function in the RS mice declined significantly.

The decline in fertility and fecundity was clearly observed in the RS group. Age-related deterioration of egg quality is widely believed to be a principal driving force behind poor pregnancy success rates in aging females. In addition, it is believed that altered ovarian steroidogenesis associated with aging brings about significant changes in uterine functions affecting implantation and a reduced capability to sustain pregnancy [41].

Menopause associated health complications negatively affect the quality of women’s lives. Decreases in gonadal steroids are believed to be the prime culprit. To explore the disorder associated with ovarian aging in the repeatedly ovulated mice, we examined bone loss and cardiac function. Our data indicated that the bone density and EF of the RS group mice were decreased compared with those of the NC group. Previous studies have indicated that lower estradiol due to diminished ovarian reserves and deteriorated follicle quality contributed to osteoporosis and decreased cardiac function [42,43].

Many women have to receive several occasions of superovulation to become pregnant throughout their treatment process. Researchers have reported that the cumulative prognosis-adjusted live-birth rate across all repeated ovulation cycles continued to increase up to the ninth cycle, with 65.3% of women undergoing IVF achieving a live birth by the sixth cycle in the United Kingdom; these findings support the efficacy of extending the number of IVF cycles beyond 3 or 4 [15]. In some countries or regions, such as Israel, many women may even have 12-20 repeated ovulations due to insurance policies [16]. Is there a limit to the number of repeated ovulation cycles for an individual patient? In this article, we discussed long-term RS with a focus on changes in ovary and heart function and bone density. Although generally safe, our results suggest that repeated ovulation does carry the risk of significant complications. In our study, RS not only decreases ovarian reserves and reduces ovarian function but also increases the risk of osteoporosis and cardiovascular disease. Awareness of the potential for these complications combined with adjustments in techniques and protocols can help reduce the rates of complications and ensure the safety of the procedures. Continued research is needed to ensure that morbidity from superovulation is minimized.

## MATERIALS AND METHODS

### Ethics statement

The experiments were approved by the Animal Care Committee of the Tongji Medical College at the Huazhong University of Science and Technology in China.

### Repeated superovulation procedures

Forty-eight specific pathogen-free female C57BL/6J mice (6 weeks old) were obtained from Beijing HFK Bio-Technology Co., LTD. (Beijing, PR China). The mice were housed at a controlled temperature and allowed free access to rodent diet and water. Sixty mice were randomly divided into two groups: normal control (NC) group and RS group.

Mice of the RS group were injected intraperitoneally with 5 IU PMSG at 13:00 (Hangzhou Animal Medicine Factory, Hangzhou, PR China), followed in 48 h by injection of 5 IU hCG, also at 13:00 (Livzon Pharmaceutical Group Inc., Guangzhou, PR China). Twenty-four hours after hCG injection, the sequential administration was repeated ten times in 30 days [32]. The mice of the NC groups were injected intraperitoneally with normal saline at the same time.

### Exhaustive swimming exercise

Ten mice in each group were then removed and subjected to exhaustive swimming exercises. Water was put in a plastic pool (93×58×58 cm) until its depth reached 40 cm, and its temperature was kept at 28±1°C. Endurance was observed by compelling the mice to swim in the plastic pool, and it was measured by the time the mice moved actively in the water until they sank to the bottom of the pool without movement for 10 seconds [44,45]. After exercise, the mice were euthanized, and the ovaries were dissected and fixed in 4% paraformaldehyde for ovarian follicle counts.

### Sample collection

Mice were monitored daily during the whole treatment. Weekly, they were weighed to estimate the changes in body weight, and images were taken to observe the fur condition. Mice in both groups were euthanized by CO_2_ inhalation on the morning of metestrus after one month of treatment. Ovaries and blood samples were collected from a total of twenty mice in each group, and the ovaries were dissected and weighed for real-time polymerase chain reaction, western blotting and immunohistochemistry. The left tibias of mice were isolated for bone density analysis.

### Transmission electron microscopy

Immediately after dissection, six ovaries from each group were weighed and fixed by immersing them for 1 h in Karnovsky fixative (2.5% glutaraldehyde and 2% paraformaldehyde in 0.1 M Na-cacodylate buffer, pH 7.3), prepared as previously described [46]. Ovaries were cut into pieces approximately 1 mm^3^ using a scalpel. After dehydration in an ascending series of ethanol, the ovarian tissues were ﬁxed in osmium tetroxide and embedded in epoxy resin. The sections were studied following Reynold lead citrate staining. Small follicles with visible oocyte nuclei were randomly selected, and images of these follicles were taken using a transmission electron microscope (CM 10 Philips, Eindhoven, The Netherlands) at 80 kV.

### Ovarian follicle counts

Ten paraffin-embedded ovaries in each group were longitudinally and serially sectioned at 4 µm, and every fifth section was mounted on a glass slide, stained with hematoxylin and eosin (HE), and analyzed under a microscope by two persons who were blinded to the origin of the sections. Only follicles containing oocytes were scored by histomorphometric evaluation, as originally described [47]. The data are represented as the primordial follicle number.

### Immunohistochemistry

Eight ovaries in each group were chosen for immunohistochemistry analysis, and representative sections from each ovary were selected. Immunohistochemical analysis was used to localize 8-hydroxy-2–deoxyguanosine (8-OHdG), 4-hydroxynonenal (4-HNE) and nitrotyrosine (NTY). The ovaries were first fixed in 4% paraformaldehyde for one day, and then, they were placed into 70% ethanol and were finally bathed in paraffin. The ovaries were consecutively split into 5-μm thick slices, and each fifth section was transferred onto a slide. Immunohistochemistry for the ovary was performed by routine procedures as previously reported [48]. It is worth mentioning that the primary antibodies were diluted to appropriate concentrations (8-OHdG, ab62623, 1:100 dilution, mouse monoclonal antibody, Abcam, Cambridge, MA, Britain; 4-HNE, ab46545, 1:500 dilution, rabbit polyclonal antibody, Abcam, Cambridge, MA, Britain; NTY, ab7048, 1:50 dilution, rabbit polyclonal antibody, Millipore, Temecula, CA) and were incubated at 4°C overnight. After that, the sections were washed with PBS and then incubated with secondary antibodies and streptavidin-biotin peroxidase complex (SABC) (PK4001/PK4002, Zhongshan Golden Bridge Biotechnology, Zhongshan Golden Bridge, China). Negative controls were left to incubate with PBS rather than the primary antibody, and images were obtained with confocal microscopy (DM4000B; Leica, Germany).

### RNA isolation and real-time polymerase chain reaction

The ovaries were kept in storage at −80°C. The RNA was isolated from six ovaries per group to perform real-time polymerase chain reaction (PCR) analysis. Total RNA of the ovaries was extracted according to the manufacturer’s directions using TRIzol (Invitrogen, Carlsbad, CA, USA). The concentration of RNA was measured with the help of a NanoDrop spectrophotometer (λ=260/280 nm; ND 1000; NanoDrop Technologies, Wilmington, DE, USA). Real-time PCR was carried out using the AB StepOne Plus PCR machine (Applied Biosystems, Foster City, CA) as described in our previous study [48]. Relative gene amplification was measured by means of the delta-delta Ct method [49]. Glyceraldehyde-3-phosphate dehydrogenase (Gapdh), which is the housekeeping gene, acted as the endogenous control. The primer sequences were as follows: anti-Mullerian hormone (AMH): forward, 5'-TCCTACATCTGGCTGAAGTGATATG-3, reverse, 5'-CAGGTGGAGGCTCTTGGAACT-3'; GAPDH: forward: 5'-TGTGTCCGTCGT GGATCTGA-3', reverse: 5'-TTG CTG TTG AAG TCG CAG GAG-3'.

### Western Blotting

Ten ovaries were collected in each group, and the total expression levels of AMH and superoxide dismutase2 (SOD2) were analyzed. Tissue extracts (30 μg) were electrophoresed on a 10% SDS-PAGE gel and transferred to polyvinylidene fluoride membranes. The membranes were blocked using 5% nonfat milk in Tris-buffered saline (10 mM Tris, 150 mM phosphate buffered saline, pH 8.0) for 1 h at room temperature. Incubation of the membranes was then performed overnight at 4°C with appropriately diluted primary antibodies (AMH, AF1446, 1:250 dilution, goat antibody, R&D Systems, Minneapolis, USA; SOD2, ab68155, 1:1000 dilution, rabbit monoclonal antibody, Epitomics, California, USA; Actin, 1:1000 dilution, rabbit polyclonal antibody, Boster, China). The blots were subsequently incubated with secondary antibodies (1:1000 dilution). The immunoreactive bands were observed using alkaline phosphatase (ALP) and BCIP/NBT staining, and finally, every single blot was performed three more times.

### Enzyme immunoassay for 17β-estradiol and progesterone

Concentrations of 17β-estradiol and progesterone in serum were determined using an enzyme linked immunosorbent assay (EIA) kit (Cayman Chemical Company, Ann Arbor, USA) according to the manufacturer's instructions. Briefly, 50 μl of the serum samples was incubated in microtiter wells that were coated with goat anti-mouse monoclonal antibodies against estradiol or progesterone. After incubation and washing, concentrations of 17β-estradiol and progesterone were determined using a spectro-photometer (Bio-Tek, Winooski, USA).

### Estrous cycling and fertility status tests

The remaining ten mice in each group were used for estrous cycling and fertility status tests. Estrous cycling in individually housed mice was evaluated every morning for twenty days using vaginal cytology, and mating trials were initiated twenty days after RS treatment and lasted for six months for all of the remaining mice. Males were randomly rotated through the cages during the mating trials. Once pregnancy was detected, the animals were placed in separate cages, and after delivery, these females were subsequently placed back into the mating cage for the next mating. When mating trials were over, all mice were euthanized using CO_2_ inhalation.

### Ultrasound Imaging and Doppler Echocardiography

Ultrasound scanning was conducted with a Vevo®1100 Imaging system on six mice from each group after six months of mating. The mice were sedated using 1.5% isoflurane, with the body temperature maintained at 36–37°C and a heart rate of 450–550 beats per minute (bpm). The B-mode and M-mode axial resolutions were 0.05–0.1 mm, and the B-mode lateral resolution was 0.2–0.5 mm [50]. The temporal frame rate in echo-mode was set at 60 Hz. A 1.0-mm sampling gate was used to obtain inflow and outflow velocities, and the maximal sweep speed was 200 mm/sec. The spectral Doppler and M-mode images were recorded in multiple screen shots, with the Doppler and M-mode measurements obtained from individual heart beats. Ultrasound parameters acquired from spectral Doppler, 2D and M-mode imaging will be introduced in the results.

### Bone density measurements

Ten mid-shaft tibias from each group were used for bone density analysis. The bone mass analysis using mouse micro-CT was carried out with a SCANCO µCT50 (SCANCO Medical). The scans were performed using instrument settings as follows: E=55 KVp, I=110 µA, increment 7.4 µm, threshold value=375.

### Statistical analysis

Statistical evaluations were performed using SPSS 17.0 software. Differences in body weight, estrous cycles, exhausted swimming time, hormones, bone density, and cardiovascular ultrasound parameters were analyzed using Student’s t test. Differences in 8-OHdG, 4-HNE and NTY expression between the two groups were analyzed using the non-parametric Kruskal–Wallis test or the Mann–Whitney test as applicable. Values represent the mean ± SEM, and P<0.05 was considered significant.
