# Correction: Repeated superovulation increases the risk of osteoporosis and cardiovascular diseases by accelerating ovarian aging in mice

**DOI:** 10.18632/aging.101569

**Published:** 2018-09-22

**Authors:** Jinjin Zhang, Zhiwen Lai, Liangyan Shi, Yong Tian, Aiyue Luo, Zheyuan Xu, Xiangyi Ma, Shixuan Wang

**Affiliations:** 1 Department of Obstetrics and Gynecology, Tongji Hospital, Tongji Medical College, Huazhong University of Science and Technology, Wuhan, Hubei, People’s Republic of China; 2 Maternal and Child Health Hospital of Zigong, Sichuan, People’s Republic of China; 3 Department of Obstetrics and Gynecology, Hubei Maternity and Child Health Care Hospital, Wuhan, Hubei, People’s Republic of China; 4 The Central Hospital of Enshi Autonomous Prefecture, Enshi Autonomous Prefecture, Hubei, People’s Republic of China

Original article: Aging (Albany NY) 2017; 10: 1089-1102

**This article has been corrected:** The authors have submitted the wrong composite Figure 6 (D) which had employed SEM photographs of other group mice unintentionally. The corrected Figure 6 is provided below. The authors declare that this correction does not change the results or conclusions of this paper. The authors sincerely apologize for this error.

**Figure 6:**
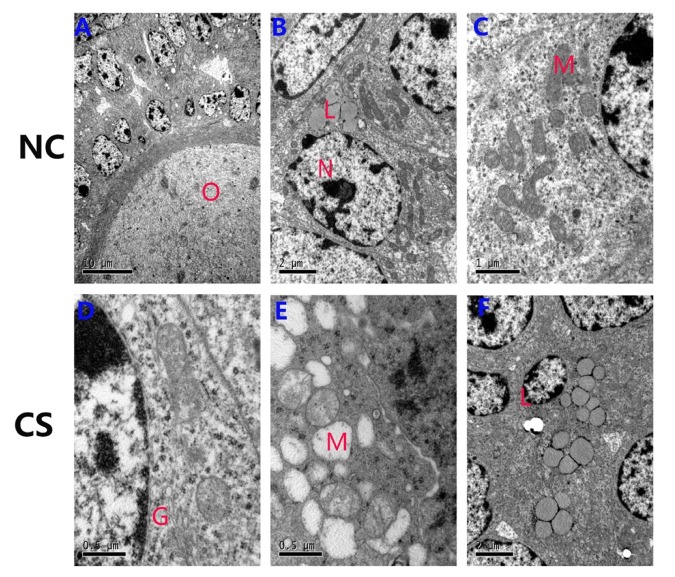
**Ultrastructural changes of the ovarian granulosa cells in both groups.** (**A-C**) The normal ultrastructure in the ovaries of NC mice. Few lipids were observed in the ovaries, and most mitochondria structures were normal. (**D-F**) The mitochondria were swollen, exhibited decreased matrix density, and developed flocculent dense bodies in the matrix space in the RS group mice. The number of lipid droplets and swollen Golgi complexes were increased in RS group mice. (O: oocyte; N: nucleus of granulosa cells; M: mitochondrial; L: lipid; G: Golgi complex).

